# Symptomatic Cavum Septum Pellucidum Cyst: A Rare Presentation

**DOI:** 10.7759/cureus.10395

**Published:** 2020-09-11

**Authors:** Bharat Pillai, Umar Farooque, Mikki Sapkota, Syed Adeel Hassan, Laszlo L Mechtler

**Affiliations:** 1 Neurology, Amrita Institute of Medical Sciences, Kochi, IND; 2 Neurology, Dow University of Health Sciences, Karachi, PAK; 3 Neurology, Geisinger Medical Center, Philadelphia, USA; 4 Internal Medicine, Dow University of Health Sciences, Karachi, PAK; 5 Neurology, Neuroradiology and Headache Medicine, Dent Neurologic Institute, Buffalo, USA

**Keywords:** intracranial cyst, cavum septum pellucidum, headache, hydrocephalus, positional headache, csf fluid dynamics, endoscopic repair, refractory headache, cyst fenestration, neuroendoscopy

## Abstract

A cavum septum pellucidum is a cerebrospinal fluid (CSF) filled cavity situated between the lateral ventricles and is considered as a normal anatomic variant sporadically seen on neuroimaging. While a cavum septum pellucidum is a relatively uncommon incidental neuroimaging finding, symptomatic cysts of the cavum septum pellucidum are very rare, with only a few cases reported in the literature so far. They are defined as fluid-filled structures with lateral bowing of the walls and membranes separated by at least 10 mm or more. We present the case of a 25-year-old male patient with a rapidly expanding cyst of the septum pellucidum with headaches refractory to conventional pharmacological therapy. A 3T magnetic resonance imaging (MRI) of the brain with contrast was performed, which confirmed the diagnosis. Due to the failure of non-interventional treatment, he was treated with therapeutic endoscopic fenestration of the cyst. Postoperatively, he reported a complete resolution of the presenting symptoms.

## Introduction

Cavum septum pellucidum (CSP) cyst, cavum vergae, and cavum velum interpositum are various presentations of benign midline anterior intracranial cysts. They are pathological when symptomatic, which arise depending upon the size of the cysts. Cavum septum pellucidum cysts are rare lesions with an incidence of 0.04% [1]. Symptomatic cysts of CSP are even rarer, with only a few cases reported in the literature. A CSP cyst can present with a myriad of symptoms, including headache, focal neurological deficits, ataxia, seizures, papilledema, emesis, syncope, visual, and even sensorimotor findings. Gradually expanding cysts may even present with visual, behavioral, or autonomic symptoms. While the majority of the CSP cysts are incidental findings, they tend to remain asymptomatic even with mass effect. Headache, however, is the most common symptom, and a causal association to the cyst becomes a diagnosis of exclusion.

## Case presentation

We present the case of a 25-year-old right-handed male who presented with daily headaches for three weeks, which actually have been present and have been progressively increasing in intensity and frequency since he was 11. The pain was localized on the top of his head and the retro-orbital regions bilaterally and of a constant, dull, nagging nature. It was made worse with coughing, sneezing and straining, and relieved with lying down and sleep. Over the past five years, the headaches have been constant, present through the day, and have been worsened by looking up and upon standing up. He has also had difficulty concentrating. There was no history of visual changes, or changes in sleep, seizures, weakness, unsteadiness, fainting, or falls. He had been treated for tension headaches and migraines for over the same time at various clinics to no lasting effect. A cavum septum pellucidum cyst and cavum vergae were noted on a computed tomography (CT) scan during the evaluation of the headache at an outside clinic in 2015. 

On presentation to our clinic, the examination was unremarkable with stable vitals and no focal neurological deficits.

**Figure 1:**
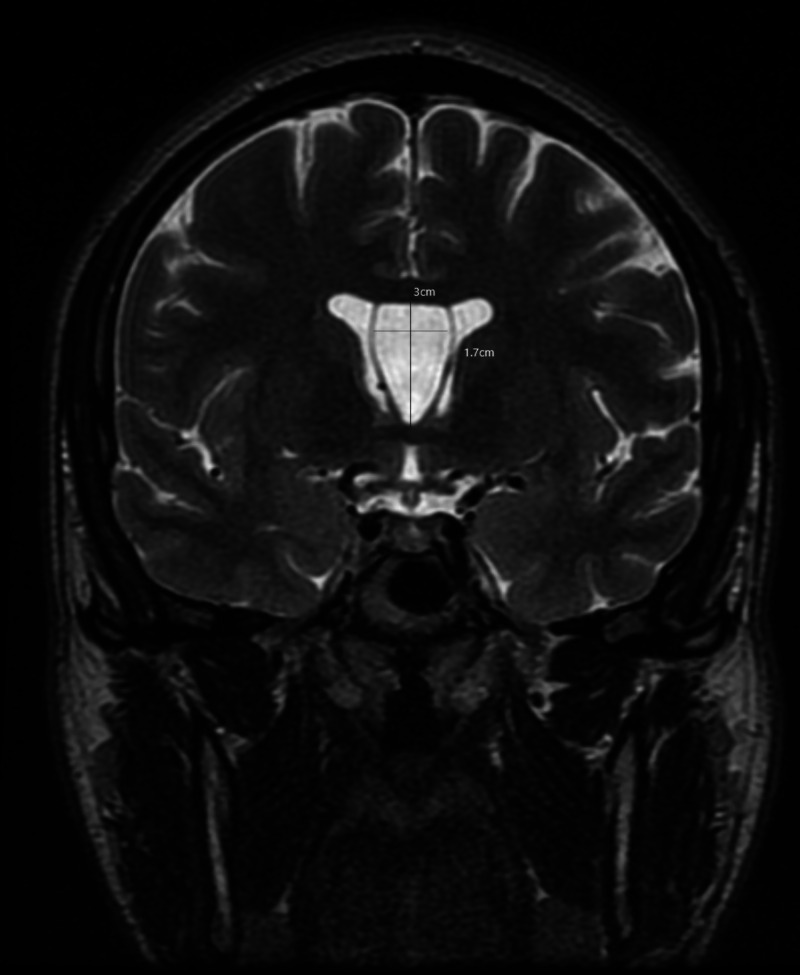
T2 weighted magnetic resonance imaging (MRI) of the head in coronal view This is a T2 weighted magnetic resonance imaging (MRI) of the head in coronal view that is showing the full longitudinal extent of the cyst of the cavum septum pellucidum et vergae. Lateral bowing of the walls of the cavum is clearly visible which is one of the criteria required to diagnose a cyst of the cavum septum pellucidum.

**Figure 2:**
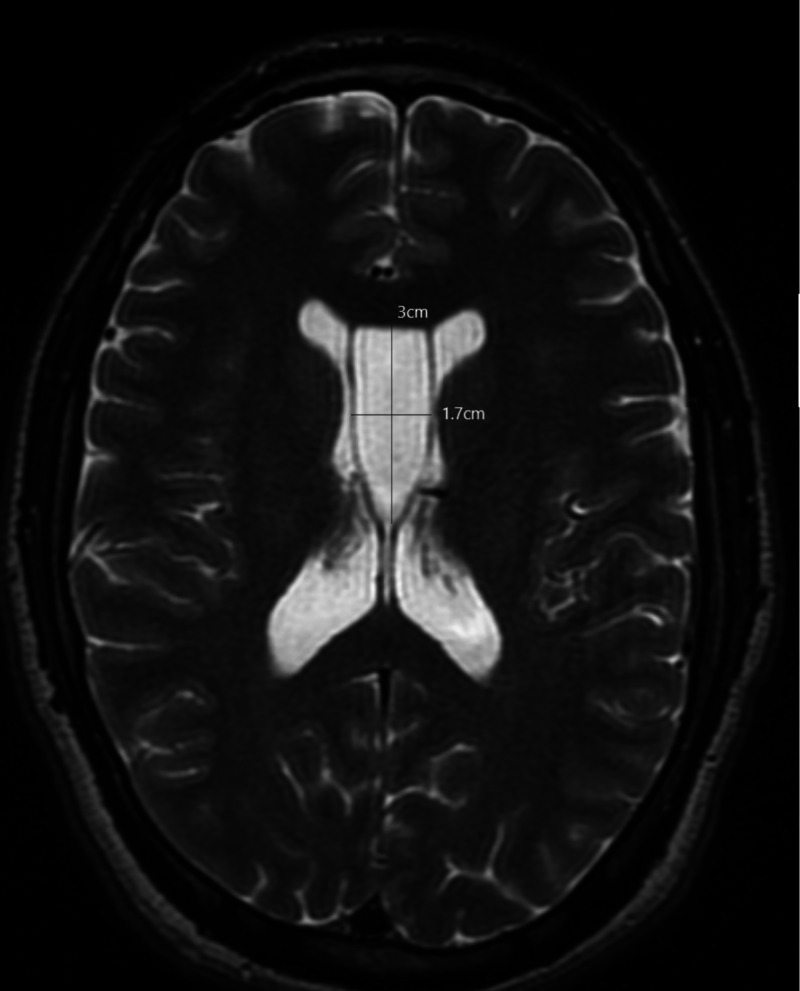
T2 weighted magnetic resonance imaging (MRI) of the head in axial view This is a T2 weighted magnetic resonance imaging (MRI) of the head in axial view that is showing the anteroposterior extent of the cyst of the cavum septum pellucidum along with the cavum vergae. The 3 X 1.7 cm cyst is appreciable in this section with definite bowing of the lateral walls.

A magnetic resonance imaging (MRI) of the brain with contrast was ordered, which revealed a cavum septum pellucidum cyst and cavum vergae. The 3 X 3 X 1.7 cm cavum septum pellucidum cyst was seen compressing the frontal horn mid bodies of lateral ventricles. The lateral ventricles were mildly enlarged bilaterally. Sections through the foramina of Monro revealed no definite communication from the cyst to the right lateral ventricle or the third ventricle. A minimal connection was appreciated to the left lateral ventricle. Partial luminal obstruction at the level of the left foramen of Monro was appreciated. There was no evidence of hydrocephalus or midline shift. An appreciable increase in size in cavum septum pellucidum from compared to a previous scan from 2015, from 1.3 X 0.9 cm to its current dimensions of 3 X 3 X 1.7 cm, was observed (Figures 1, 2).

Due to the failure of pharmacological therapy, the headaches were considered to be related to the expanding CSP cyst. A neurosurgery consult was sought, and the patient proceeded to surgery via endoscopic fenestration of the cyst. Following surgery, the patient was seen at eight weeks post-op at our clinic and reported a complete resolution of the presenting symptoms, thus supporting our hypothesis.

## Discussion

The septum pellucidum (SP) is a thin, triangular, double membrane with the apex at the foramen of Monro and base towards the quadrigeminal cistern separating the frontal horns of the right and left ventricles. CSP is a potential cavity between the two leaves of septum pellucidum. A CSP cyst is defined as a fluid-containing structure between lateral ventricles at least 10 mm in width or with lateral bowing of the ventricular walls [2].

It is more than just a neural tract that connects the corpus callosum and fornix in spite of the misconception that the septum pellucidum has no function (based on the poor understanding of its embryology and connections). It is a component of the limbic system that acts as a relay station to the main hippocampal and hypothalamic nuclei, as is evidenced by symptoms of mental retardation and learning disabilities seen with septum pellucidum lesions [2-4].

The septum pellucidum is bounded superiorly by the inferior surface of the body of the corpus callosum; anteroinferior by the superior surface of the genu of the corpus callosum; posteroinferior by corpus and columns of the fornix; laterally by the medial wall of the frontal horns of the lateral ventricles and medially by the virtual space between the contralateral septum.

The cavum septum pellucidum is a normal anatomic variant, is usually asymptomatic and discovered incidentally. Cavum vergae is the term assigned to the posterior extension of the CSP.

In more than 85% of cases, the CSP fuses by three to six months of life. The prevalence of CSP sharply declines soon after delivery, such that it is 85% at one month, 45% at two months, and 15% at three to six months. It closes in a caudal to rostral fashion, resulting in obliteration of the caudal portion first (at 38 weeks of gestation). The anterior portion only obliterates by three to six months of age, which occurs due to the rapid growth of the corpus callosum and hippocampal alvei along with the concrescence of cerebral hemispheres leading to the fusion of the membranes of SP. Cavum vergae is present in around 30% of newborns and persists in only 1% of adults. Formerly, SP and CSP were known as fifth and sixth ventricles, respectively. However, since the cyst cavity is not continuous with the ventricular system and lacks the choroid plexus, this nomenclature is no longer used [2-6].

Depending on its communication with the ventricles, CSP cysts are classified as communicating and non-communicating. Secondary communications may be formed by head trauma, surgery, or spontaneous rupture. CSP cysts may also be classified as asymptomatic (incidental) or symptomatic (pathological, non-communicating cavum with increased pressure within the cyst) as proposed by Shaw and Ellsworth [7,8].

Headache is the most common and consistent symptom associated with CSP cysts and is thought to be due to intermittent hydrocephalus provoked by positional changes and other maneuvers that cause a rise in the intracranial pressure (Valsalva, straining, etc.). CSP cysts constitute a rare but extremely important cause of positional headaches that are potentially reversible. The differential diagnosis for anterior intracranial midline cysts includes asymmetric lateral ventricle cysts where the septum pellucidum is bowed but intact, the vein of Galen aneurysm, cavum septum arachnoid cyst, and an interhemispheric cyst related to agenesis of corpus callosum and cavum velum interpositum [9-11].

## Conclusions

Although rare, a cyst of cavum septum pellucidum should be included in the differential diagnosis when a patient presents with symptoms attributable to obstruction to cerebrospinal fluid (CSF) flow. The preferred neuroimaging modality is with a 3T MRI with contrast, which clearly shows a large intracranial midline interhemispheric cyst, along with delineating the extent and its communications with either lateral or the third ventricles. We present a rare case of enlarged symptomatic cyst of cavum septum pellucidum, presenting with episodic positional headaches not responding to pharmacological therapy. Complete resolution of the symptoms was seen after endoscopic fenestration of the cyst, establishing the correlation between headache and the cyst of cavum septum pellucidum.
